# Supplementary material for: Content-rich biological network constructed by mining PubMed abstracts
Source: BMC Bioinformatics. 2004 Oct 8;5:147. doi: 10.1186/1471-2105-5-147 (PMC528731; doi:10.1186/1471-2105-5-147)
Supplement: Additional File 5 — The original Chilibot query results of the term "long-term potentiation (LTP)" and 22 other terms, limiting the latest references analyzed to the years 1990, 1995, 2000, and 2004. [file 1471-2105-5-147-S5.bz2 › chilibotAdditionalFile5/ltp2004/html/PI-3K_CAMKII.html]

 


 **PI-3K** and **CAMKII** 
  
Found 6 abstracts in PubMed,  **6 abstracts were retrieved and analyzed**.  


---

 Search Google  |
 PDF files only 
|  EDU domain only 

---

**Interactive relationship** (e.g. stimulation, inhibition, etc)

**Parallel relationship** (e.g. studied together, co-existance, homology, etc.)

- These findings led us to hypothesize that depolarization mediated survival occurs through both the PI 3 kinase  [ **PI-3K** ]  Akt and the  **CaMKII**  pathways.  Ref: 10825497 Brain Res, 2000
- Wortmannin is a potent inhibitor of phosphatidylinositol PI 3 kinase  [ **PI-3K** ]  and PI 3 kinase  [ **PI-3K** ]  related proteins e.g. ATM, but it does not inhibit the activity of purified calmodulin dependent protein kinase II  **CaMKII** .  Ref: 10576651 Br J Cancer, 1999
- It is thus likely that PI 3 kinase  [ **PI-3K** ]  and  **CaMKII**  independently promote the membrane depolarization mediated survival of sympathetic neurons in culture.  Ref: 10825497 Brain Res, 2000
